# Supplementary material for: Animal Protein Intake Is Associated with General Adiposity in Adolescents: The Teen Food and Development Study
Source: Nutrients. 2019 Dec 31;12(1):110. doi: 10.3390/nu12010110 (PMC7019331; doi:10.3390/nu12010110)
Supplement: Supplementary file 1 [file nutrients-12-00110-s001.pdf]

Table S1. Categories of protein foods, their descriptions, and corresponding FFQ food items

Protein Food Category	Description	FFQ Food Items
**Red Meat**	Red meat	Red meats (e.g. beef, lamb, etc); red meat patties, steak, and meatloaf; red meat in hamburgers, sandwiches, burritos, taco, quesadilla, pizza, spring rolls, nachos; in mixed dishes such as vegetable dishes, pasta, rice dishes, legume dishes.
**White meat**	White meat/Poultry	Chicken; chicken patties, meatloaf or meatballs, nuggets; chicken or turkey in sandwiches, sandwiches, burritos, taco, quesadilla, pizza, spring rolls, nachos; in mixed dishes such as vegetable dishes, pasta, rice dishes, legume dishes
**Processed Meat**	Processed white or red meat	Links and franks/frankfurters, bacon, hotdogs in sandwiches; corndogs
**Fish**	Fish	Fish (all kinds); fish in burritos, tacos, and sushi/kimbap
**Dairy**	Dairy, whey and casein	Cow's milk or goat's milk, cheese (all types or in cheeseburgers, sandwiches, burritos, taco, quesadilla, pizza, nachos), yogurt (all kinds), Ice cream (all kinds, sorbet or sherbet), cow's milk-based beverages (smoothies, shakes, frapuccino and lattes) and cream/sour cream/cream based sauces/cream based soups; whey and casein protein isolate in meat analogues
**Egg**	Egg	Egg (any style) in egg salads, sandwiches, tacos, spring rolls, vegetarian sushi/kimbap
**Grain**	Grains/Cereals	Bread (all kinds), pasta/noodles, rice, cereals (all kinds/cold and hot, including granola and breakfast bars), and pancakes
**Gluten**	Gluten, gluten extracts	Gluten and gluten extract in meat analogues
**Soy**	Soybeans, soy beverages, soy extracts, soy dairy substitute	Tofu, tempeh, edamame, soy milk (all kinds) and soy milk-based beverages (smoothies, shakes, lattes), soy ice cream, soy yogurt, tofu soup, soy nuts
**Non-Soy Legumes**	Non-soy legumes or beans	Beans (all kinds) in meatless burgers/patties, burritos, tacos, nachos, , haystack; hummus; mixed bean soup, lentil/split pea soup
**Nuts**	Nuts, seeds, nut butters	Nut and nut butters; peanut butter in peanut butter sandwich
